# UGT1A1 gene and neonatal hyperbilirubinemia: a preliminary study from Bengkulu, Indonesia

**DOI:** 10.1186/s13104-018-3284-y

**Published:** 2018-03-13

**Authors:** Radhian Amandito, Raihandhana Putradista, Clara Jikesya, Dwi Utaminingsih, Jumnalis Rusin, Rinawati Rohsiswatmo, Amarila Malik

**Affiliations:** 1 M Yunus General Hospital, Jl Bhayangkara, Bengkulu City, Bengkulu Indonesia; 2 Neonatal Intensive Care Unit, Pondok Indah General Hospital, Jl Metro Duta Kav. UE, Pondok Indah, Jakarta, Indonesia; 3 Division of Pharmaceutical Microbiology and Biotechnology, Faculty of Pharmacy, Universitas Indonesia, UI Depok Campus, Depok, West Java 16424 Indonesia; 4 Department of Pediatrics, M Yunus General Hospital, Jl Bhayangkara, Bengkulu City, Bengkulu Indonesia; 5 Division of Perinatology, Department of Pediatrics, Cipto Mangunkusumo General Hospital, Faculty of Medicine, Universitas Indonesia, Jl Pangeran Diponegoro No. 71, Central Jakarta, Jakarta, Indonesia

**Keywords:** Bilirubin, Glucuronosyltransferase, Indonesia, Neonatal hyperbilirubinemia, Restriction fragment length polymorphism

## Abstract

**Objective:**

The genetic involvement in unconjugated neonatal hyperbilirubinemia has been extensively studied. Despite the high incidence of hyperbilirubinemia in Indonesia, studies are lacking. The objective of this study is to elucidate the role of polymorphism in the *UGT1A1* in Neonatal Hyperbilirubinemia in Bengkulu, Indonesia.

**Results:**

There were 41 neonates enrolled in the study; 30 had a total serum bilirubin level ≥ 15 mg/dL (hyperbilirubinemia neonates) while 11 has < 15 mg/dL (control neonates). Genetic mutations in Exon 1, UGT1A1*6 (c211g > a) and one in promoter region, UGT1A1*60 (c3279t > g) were determined by polymerase chain reaction–restriction fragment length polymorphism. We found 18 (60%) mutation in exon 1 in hyperbilirubinemia group and 7 (64%) in the control group with an identical allele frequency of 0.3 in both groups. We found heterozygous UGT1A1*60 4 times (13.3%) and homozygous 26 times (86.7%) in the hyperbilirubinemia group, with an identical allele frequency of 0.935 in hyperbilirubinemia and 1 in control group. This study supports the involvement of genetic factors in the development of unconjugated hyperbilirubinemia in Bengkulu population.

## Introduction

Neonatal hyperbilirubinemia is a common condition in neonates caused by the combination of increased heme catabolism and physiologic immaturity of the liver in bilirubin conjugation and excretion [1]. This condition may lead to kernicterus, which causes disability and death [2]. Risk factors for hyperbilirubinemia have been determined, albeit with varying degrees. They range from ABO incompatibility, premature birth, breastfeeding, to genetic conditions such as G6PD deficiency and polymorphisms in *UGT1A1* [3].

UDP-glucoronosyltransferase 1A1 (UGT1A1) is the key enzyme for bilirubin conjugation. It is mapped to the chromosome 2q37 [4]. A variety of allelic polymorphisms of human *UGT1A1* have been reported, with some leading to partial or complete deficiency of enzyme activity [5].

The UGT1A1*6 (c211G > A) mutation in exon 1, which causes decreased enzyme activity, was found to be associated with neonatal hyperbilirubinemia in Asian populations, as well as one of the most prevalent polymorphism in East Asian population [6]. The UGT1A1*60 (c-3279T > G) mutation in promoter region was found to be a frequent mutation in the Egyptian neonates with neonatal hyperbilirubinemia [7].

Despite a study by Sutomo on Indonesian population, study on different ethnic populations of Indonesia for polymorphism of the *UGT1A1* gene is needed [8]. In the ethnic group of Indonesia in Bengkulu, a high number of neonatal hyperbilirubinemia was reported by the Provincial Referral Hospital in the year 2016; 80 out of 362 neonates treated in the NICU. In addition, Bengkulu with an area of approximately 20,000 km^2^ and a population of fewer than 2 million people is isolated in terms of ethnic diversity, making it an ideal location to study ethnic-related polymorphism. To gather more information on *UGT1A1* gene variation in Indonesia, we investigated the association between UGT1A1*60 and UGT1A1*6 in Bengkulu neonates.

## Main text

### Materials and methods

#### Study population

This cross-sectional study was conducted in neonatal intensive care unit of M. Yunus General Hospital, Bengkulu, Indonesia. The study was approved by the Health Research Ethics Committee—University of Indonesia and national referral hospital Cipto Mangunkusumo, Jakarta. Data collection was conducted from November 2016 to February 2017. A written informed consent was obtained from the parents before inclusion into the study. Sample size calculation was done by Total Population Sampling due to the low number of population available within the set amount of time. The study population included 30 infants with hyperbilirubinemia (cases) and 11 infants as controls, which includes all eligible population. Both preterm and term neonates were assessed for eligibility.

Hyperbilirubinemia was determined from the total plasma bilirubin (TPB) of neonates patient obtained by using Bilistick [9] between 3 and 7 days of birth. Neonates were divided according to TPB in high risk hyperbilirubinemia (≥ 15 mg/dL) or low risk, control infants (< 15 mg/dL) [10]. Neonates with evidence of hemolysis (fall in Hemoglobin), phototherapy, blood transfusion or exchange transfusion, sepsis, asphyxia, cephalhematoma, and major congenital malformations were excluded. All neonates were recorded in terms of sociodemographic and clinical variables through clinical examination and laboratory investigations, as well as a review of the patient’s medical record for previously obtained data of birth records.

#### Laboratory investigations

We performed blood examination. TPB was obtained by Bilistick device [9] and hemoglobin, hematocrite, platelets, and leukocyte count with standard lab technique. In addition, 1 mL of venous blood was collected in a vial containing EDTA for DNA extraction. Prior to DNA extraction, the blood samples were stored at − 20 °C. All DNA samples were analyzed for mutations in the promoter region and exon 1 of UGT1A1 gene by polymerase chain reaction–restriction fragment length polymorphism (PCR–RFLP) method [11].

#### Genomic DNA extraction and polymerase chain reaction

Genomic DNA was extracted from both blood samples by using QIAamp DNA Blood Mini Kit (QIAGEN, Germany) according to the manufacturer. Genomic DNA as much as 2 ml was added to a PCR mix containing 34.5 μl dH_2_O, 5 μl 10X KOD polymerase buffer, 4 μl 25 mM MgSO_4_, 7 μl 2 mM dNTPs (dATP, dCTP, dGTP, dTTP), and 0.7 μl KOD polymerase (Novagen, Germany). PCR amplification consisted of an initial denaturation of 2 min at 95 °C followed by 30 cycles of denaturation of 94 °C for 30 s, annealing at 55 °C for 15 s, extension at 72 °C for 1 min, post extension at 72 °C for 5 min and hold at 4 °C for 60 min. PCR reactions were carried out in a PCR thermocycler (Biometra, Germany). All PCR products were analyzed on 2% Ethidium bromide (EtBr) containing agarose gel in 1 x TAE buffer, whereas for the detection of c-211G > A mutation on the Exon 1 and c-3279T > G mutation on the promoter region of UGT1A1 gene, 3% EtBr gel in 1 x TBE were used.

#### Detection of UGT1A1*6

We used the restriction enzyme AvaII (NEB, USA) and amplified the exon 1 of *UGT1A1* gene by using primers UGT1A1_exon1Fw (U1F1 forward) 5′-AGATACTGTTGATCCCAGTG-3′ and UGT1A1_exon1Rv (U211R reverse): 5′-CTTCAAGGTGTAAAATGCTC-3 according to a previous study [12].

#### Detection of UGT1A1*60

We used the restriction enzyme DraI (NEB, USA) and amplified the promoter region of *UGT1A1* gene by using primers UGT1A1_promoter Fw (forward) 5′-CAC-CAGAACAAACTTCTGAG-3′ and UGT1A1_promoter Rv (reverse) 5′-CTGTCCCTTCTGAAT-CATTG-3′ to detect the mutation c-3279T > G according to the primer reported in a previous study [8].

#### Statistical analysis

Categorical independent variables and the categorical dependent variable were analyzed using Fishers exact test, while numerical dependent variables and categorical dependent variable were analyzed using student’s t-test to see the association between the independent variables and dependent variable. Categorical variables were presented as percentages (sex, Breastfeeding, delivery method, sibling history of hyperbilirubinemia requiring phototherapy, primipara mother, race, c211G > A mutation, and c-3279T > G mutation), while numerical variables were expressed as mean and range (Gestational age, birth weight, and total plasma bilirubin). The software STATA ver 12 for macOS was used.

### Results

#### Demographic and clinical characteristics

Forty-one term and preterm infants with total plasma bilirubin concentration of ≥ 15 mg/dL (high risk) or < 15 mg/dL (low risk) were enrolled. The clinical characteristics of the patients are reported in Table 1. The peak bilirubin level in high risk hyperbilirubinemia was 29 mg/dL. The risk of developing high risk hyperbilirubinemia for each risk factors were estimated by either fishers exact test or student’s t test. None of the studied clinical risk factors were associated with high-risk hyperbilirubinemia; breastfeeding (OR 1.77; 95% CI 0.329–12.347), vaginal vs cesarean delivery method (OR 1.75; 95% CI 0.348–9.829), sibling history for hyperbilirubinemia requiring phototherapy (OR 0.155; 95% CI 0.003–3.491), and primipara mother (OR 2.66; 95% CI 0.497–18.229). These findings are in line with a study that suggested not all neonates with hyperbilirubinemia have the known risk factors present [13], suggesting a different dominant risk factor is involved in different populations. Since our study was homogenous and low in samples, the tendency for our different findings was expected.

**Table 1:** Clinical characteristics of study group

Descriptive variables	Case, n(%)	Control, n(%)	OR	95% CI	*p*-value
	N = 30	N = 11			
Sex					
Male	10 (33.3%)	4 (36.4%)	0.875	0.170–5.093	1.000
Female	20 (67%)	7 (63.6%)			
Gestational age (week)					
Range	27–40	30–38	N/A	N/A	0.5290
Mean	35.4	34.6			
Birth weight (g)					
Range	1200–3700	1200–3700	N/A	N/A	0.5839
Mean	2360.66	2209.1			
Total plasma bilirubin (mg/dL)					
Range	15.2–29	8.8–14.7	N/A	N/A	0.0000
Mean	20.38	12.3			
Feeding					
Exclusive breastfeeding	18 (60%)	8 (72.7%)	1.77778	0.329–12.347	0.7158
Not exclusive breastfeeding	12 (40%)	3 (27.3%)			
Delivery method					
Vaginal	15 (50%)	7 (63.6%)	1.75	0.348–9.829	0.4993
Cesarean	15 (50%)	4 (36.4%)			
Sibling history for hyperbilirubinemia requiring phototherapy					
Yes	1 (3.33%)	2 (18.2%)	0.1551724	0.003–3.491	0.1703
No	29 (96.67%)	9 (81.8%)			
Born to primipara mother					
Yes	15 (50%)	3 (27.3%)	2.6667	0.497–18.229	0.2911
No	15 (50%)	8 (72.7%)			

Premature birth also did not show association with high-risk hyperbilirubinemia. It is known that UGT1A1 is modulated progressively according to gestational age, from 0.1% at 17–30 weeks’ gestation to 1% at 30–40 weeks’ gestation and reach adult levels by 14 weeks postnatal [14]. This shows that premature infants could aggravate the immaturity of the conjugating enzyme, thus causing a more severe hyperbilirubinemia, in addition to the exaggerated neonatal red cell, hepatic, and gastrointestinal immaturity. This suggests that despite the risk for hyperbilirubinemia, premature birth does not necessarily cause higher levels of bilirubin by itself as the risk factor.

#### Detection of *UGT1A1* mutation

Table 2 shows the distribution of *UGT1A1* variants of both the exon 1 and promoter mutation, and the allele variant. In high-risk hyperbilirubinemia neonates, the incidence of wild type (no risk for hyperbilirubinemia) was instead higher (40–36%) than UGT1A1*6 is (60–64%). While for UGT1A1*60, there was no wild type detected in either group, small incidence of heterozygous form in high risk hyperbilirubinemia (13%), and higher incidence of homozygous form in low risk hyperbilirubinemia group (87–100%). There was no association between high risk and low risk hyperbilirubinemia groups’ polymorphism status as shown by the statistical analysis.

**Table 2:** Distribution of UGT1A1 variants

Mutation	Case, n(%)	Control, n(%)
	N = 30	N = 11
UGT1A1*6		
GG	12 (40%)	4 (36%)
GA	18 (60%)	7 (64%)
AA	0 (0%)	0 (0%)
A Allele^a^	0.3	0.32
UGT1A1*60		
TT	0 (0%)	0 (0%)
TG	4 (13%)	0 (0%)
GG	26 (87%)	11 (100%)
G Allele^a^	0.935	1

^a^Ratio

#### Association between *UGT1A1* mutation and unconjugated hyperbilirubinemia

As reported in Chinese neonates [15], the possible synergistic effect of more than one mutation on TPB was investigated in our series (Table 3). The occurrence of multiple mutation varies in high risk and low risk hyperbilirubinemia groups. In high risk hyperbilirubinemia group, there is more incidence of wild type exon 1 with heterozygous UGT1A1*60 (GG/TG; 10–0%) and heterozygous UGT1A1*6 with heterozygous UGT1A1*60 (GA/TG; 3.3–0%) compared to low risk hyperbilirubinemia. While in low risk group there is more incidence of wild type exon 1 with homozygous UGT1A1*60 (GG/GG; 30–36%) and heterozygous UGT1A1*6 with homozygous UGT1A1*60 (GA/GG; 57–65%). There was no association between multiple mutation sites of *UGT1A1* and high or low risk hyperbilirubinemia.

**Table 3:** Mutation variant distribution in study group

Variation	Case, n(%)	Control, n(%)
	N = 30	N = 11
GG/TG	3 (10%)	0 (0%)
GG/GG	9 (30%)	4 (36%)
GA/TG	1 (3.3%)	0 (0%)
GA/GG	17 (57%)	7 (65%)

### Discussion

From previous studies in Javanese Indonesians and Malays, which are anthropologically closely related with Bengkulu Indonesians, the frequency of mutation of UGT1A1*6 (0.015 and 0.014) is quite low and does not differ significantly between normal bilirubin level and hyperbilirubinemia neonates [7]. This figure is similar to an Indian study with a frequency of 0.03 for hyperbilirubinemic neonates and 0.002 for healthy neonates [16]. Therefore, it can be concluded that despite being particularly frequent in East Asian population, UGT1A1*6, does not contribute to the development and/or severity of hyperbilirubinemia. In contrast to previous findings [7], our study found a relatively high incidence of UGT1A1*6 mutation in Bengkulu Indonesian population in both hyperbilirubinemia and normal bilirubin neonate, compared to the frequency in other populations (0.3 and 0.32 respectively). This discrepancy could be accounted by the more homogenous ethnicity and isolated population of Bengkulu and by a difference in sample size. Despite the high prevalence of the mutation, it is not a specific risk factor to the development of high risk neonatal hyperbilirubinemia with a high level of bilirubin in Bengkulu population.

UGT1A1*60 decreases the transcriptional activity of *UGT1A1* by 60% [17]. A study on Malaysian neonates [18] concluded that the allele frequency of UGT1A1*60 was significantly higher in hyperbilirubinemia neonates when compared to controls. Between the homozygous and heterozygous group of case and control, the heterozygous cases were not significantly different compared to the heterozygous controls, suggesting that presence only of the UGT1A1*60 does not per se cause neonatal hyperbilirubinemia, but rather is a risk factor to this condition. Similar findings were reported in Egyptian neonates [8]. The allele frequency of UGT1A1*60 in Egyptian neonates was 49.2% for the hyperbilirubinemia group and 25.6% for the control group, while in East Asian neonates the frequency is lower ranging from 25% [19] to 27% [20].

Our findings are in line with the ethnically similar population of Malaysian neonates [18] and the Egyptian [8], with allele frequency of 0.935 in high risk hyperbilirubinemic neonates and 1 in controls. Despite the high frequency between both groups, the difference is not statistically significant, even for the high frequency in the control group. This suggests that mutation in the promoter region alone does not cause high risk hyperbilirubinemia in Bengkulu neonates.

It has been previously shown that not one, but several mutations are involved in causing the non-physiologic hyperbilirubinemia in neonates [15, 16]. In contrast to previous studies, no specific combination of risk factor were observed even with the combination of the 2 highest risk mutation variant found in our group (GA/GG). This data suggests a different combination of clinical and genetic factors for Bengkulu neonates compared to other races.

Although hyperbilirubinemia is common, in Indonesia, the number of kernicterus cases reported are rather small. In addition, the causes of these severe cases are unknown. Accordingly, a study into other possible etiologies including genetic risk factors in Indonesian neonatal hyperbilirubinemia is necessary.

### Conclusion

In Bengkulu neonates, both mutation UGT1A1*6 and UGT1A1*60 was found in hyperbilirubinemic neonates but they do not act as individual risk factors to the severity of the neonatal hyperbilirubinemia. Therefore, the high incidence of neonatal hyperbilirubinemia in Bengkulu is not solely caused by UGT1A1*6 nor UGT1A1*60 polymorphism. Further studies are necessary with more sample size and inclusion of other polymorphisms.

## Limitations

We limited our study to two mutations that are commonly found in Asian neonates. We also used only neonates of Bengkulu descendant, with no comparison with non-Bengkulu Indonesians. The available population from the set of time determined was low thus statistical bias was possible.

## Abbreviations

TPB: total plasma bilirubin
PCR–RFLP: polymerase chain reaction–restriction fragment length polymorphism
UGT1A1: UDP-glucoronosyltransferase 1A1
